# Wheat leaf rust fungus effector Pt13024 is avirulent to TcLr30

**DOI:** 10.3389/fpls.2022.1098549

**Published:** 2023-01-16

**Authors:** Yue Qi, Jianyuan Li, Johannes Mapuranga, Na Zhang, Jiaying Chang, Qianhua Shen, Yue Zhang, Jie Wei, Liping Cui, Daqun Liu, Wenxiang Yang

**Affiliations:** ^1^ Department of Plant Pathology, Agricultural University of Hebei/Technological Innovation Center for Biological Control of Plant Diseases and Insect Pests of Hebei Province/National Engineering Research Center for Agriculture in Northern Mountainous Areas, Baoding, China; ^2^ Key Laboratory of Plant Resources, Institute of Botany, Chinese Academy of Sciences, Beijing, China; ^3^ College of Biological Sciences and Engineering, Xingtai University, Xingtai, China; ^4^ Institute of Genetics and Developmental Biology, Chinese Academy of Sciences, Beijing, China; ^5^ Dryland Farming Institute, Hebei Academy of Agricultural and Forestry Science, Hengshui, China; ^6^ Department of Agriculture and Animal Husbandry Engineering, Cangzhou Technical College, Cangzhou, China

**Keywords:** *Puccinia triticina*, effector, wheat rust, host–pathogen interaction, biotroph, pathogenicity

## Abstract

Wheat leaf rust, caused by *Puccinia triticina* Eriks. (*Pt*), is a global wheat disease threatening wheat production. Dissecting how *Pt* effector proteins interact with wheat has great significance in understanding the pathogenicity mechanisms of *Pt*. In the study, the cDNA of *Pt* 13-5-72 interacting with susceptible cultivar Thatcher was used as template to amplify Pt13024 gene. The expression pattern and structure of *Pt13024* were analyzed by qRT-PCR and online softwares. The secretion function of Pt13024 signal peptide was verified by the yeast system. Subcellular localization of Pt13024 was analyzed using transient expression on *Nicotiana benthamiana*. The verification that Pt13024 inhibited programmed cell death (PCD) was conducted on *N. benthamiana* and wheat. The deletion mutation of Pt13024 was used to identify the virulence function motif. The transient transformation of wheat mediated by the type III secretion system (TTSS) was used to analyze the activity of regulating the host defense response of Pt13024. *Pt13024* gene silencing was performed by host-induced gene silencing (HIGS). The results showed that Pt13024 was identified as an effector and localized in the cytoplasm and nucleus on the *N. benthamiana*. It can inhibit PCD induced by the Bcl-2-associated X protein (BAX) from mice and infestans 1 (INF1) from *Phytophthora infestans* on *N. benthamiana*, and it can also inhibit PCD induced by DC3000 on wheat. The amino acids 22 to 41 at N-terminal of the Pt13024 are essential for the inhibition of programmed cell death (PCD) induced by BAX. The accumulation of reactive oxygen species and deposition of callose in near-isogenic line TcLr30, which is in Thatcher background with *Lr30*, induced by Pt13024 was higher than that in 41 wheat leaf rust-resistant near-isogenic lines (monogenic lines) with different resistance genes and Thatcher. Silencing of Pt13024 reduced the leaf rust resistance of Lr30 during the interaction between Pt and TcLr30. We can conclude that Pt13024 is avirulent to TcLr30 when *Pt* interacts with TcLr30. These findings lay the foundation for further investigations into the role of Pt effector proteins in pathogenesis and their regulatory mechanisms.

## Introduction

Wheat leaf rust caused by *Puccinia triticinia* Eriks. (*Pt*), is the most widely distributed and destructive disease affecting wheat production worldwide (Rattu Ahmad et al., 2009; Savary et al., 2019). *Pt* causes both yield losses (15%–45% or even higher) and a decrease in the quality of wheat when it occurs seriously. The most inexpensive, secure, and effective approach of preventing wheat leaf rust is to use resistant cultivars. However, continuous emergence of new *Pt* races, with novel degrees of virulence, renders wheat resistance genes ineffective. Understanding the molecular pathogenesis mechanisms of *Pt* is critical for controlling wheat leaf rust.

On infection, pathogen-associated molecular patterns (PAMPs) are recognized by pattern recognition receptors (PRRs). This activates the first line of defense, also known as PAMPs-triggered immunity (PTI), to curb further colonization by the pathogen (Jones and Dangl, 2006; Jones et al., 2016). The recognition of PAMPs induces basal defense responses, which are associated with changes in the ion flux across the plasma membrane, the generation of reactive oxygen species (ROS), the deposition of callose, the activation of mitogen-activated protein kinase signaling cascades, and the expression of defense-related genes (Zipfel, 2008; Zipfel and Robatzek, 2010). To infect the host plant successfully, pathogens suppress PTI components by secreting virulence factors known as effectors through haustoria and hyphae into the host cells thereby causing diseases (Martel et al., 2021). In response to this, plants acquired a second layer of innate immunity known as effector-triggered immunity (ETI), in which plant resistance proteins recognize corresponding avirulence factors and set off a powerful defensive response (Chisholm et al., 2006; Jones and Dangl, 2006).

In recent years, studies on the functional characterization of host proteins that are targeted by effectors from bacteria and *Oomycetes* have contributed significantly to notable achievements that demonstrated that pathogen effectors regulate the host immune system by interacting with host proteins (Yang et al., 2020). Effectors can either manipulate the activity of the host targets or suppress PTI or ETI. For example, *Pseudomonas syringae* type III effectors, such as HopAI1 and HopF2, directly attack components of MAPK cascades to suppress PTI signaling (Zhang et al., 2007; Wang et al., 2010). *Puccinia striiformis* f. sp. *tritici* (*Pst*) is an effector of PstGSRE1 and suppresses host PTI-associated callose deposition and hydrogen peroxide accumulation, thereby weakening host immunity (Qi et al., 2019). PSEC2 and PSEC17 are both localized in the cytoplasm and chloroplast to inhibit the PTI response of the host (Su et al., 2021). PEC6 another *Pst* effector affects cytokinin interconversion and methyl transfer processes by targeting adenosine kinase (ADK) to promote fungal development (Liu et al., 2016). Pst12806 targets the chloroplast where it suppresses host plant basal immunity by suppressing cell death, decreasing the expression of defense-related genes and callose deposition, and causing ROS accumulation (Xu et al., 2019). Two effectors, Pst_4, and Pst_5, from *Pst* weaken wheat resistance by interacting with TaISP (wheat cytochrome b6–f complex iron–sulfur subunit, a chloroplast protein encoded by a nuclear gene) in the cytoplasm, and suppressing TaISP from entering chloroplasts, thereby limiting host ROS accumulation, and promoting fungal pathogenicity (Wang et al., 2021). The effector Pst27791 interferes with host immunity including ROS accumulation, expression of the defense genes *TaPR1/2*, and the activation of mitogen-activated protein kinase (MAPK) by targeting wheat Raf-like kinase (Wan et al., 2022). As a splicing regulator, Pst_A23 directly binds the *cis*-elements of host genes, reduce the plant defense response (Tang et al., 2022). The effector PsSpg1 indirectly interacts with the susceptibility gene *TaPsIPK1* to promote parasitism *via* enhancing the kinase activity and nuclear entry of *TaPsIPK1* (Wang et al., 2022). These studies demonstrate that pathogen effectors can manipulate the activity of many targets in host plants, and inhibit the immune defense responses of host plants by acting as enzymes and other roles.

Genome and transcriptome sequencing have become important research methodologies for exploring the function of leaf rust effector proteins. The transcriptome analysis of six *Pt* strains infecting wheat at the stage of haustoria formation found 532 candidate effector proteins and 222,571 expressed genes (Bruce et al., 2014). Another recent comparative genomics study integrated with association analysis found 20 *Pt* candidate effector proteins corresponding to *Lr20* (Wu et al., 2017). More recently, using long-read-based *de novo* genome assembly and comparative genomics of the wheat leaf rust pathotypes Pt104, 38, 31, and 37 candidate avirulence genes to *Lr26*, *Lr2a*, and *Lr3ka*’) respectively, were identified (Wu et al., 2020). However, the mechanisms by which wheat leaf rust effector proteins interact with their host targets are still not clear. Pt3 and Pt27 were postulated as two candidate avirulent effectors of *Lr9*, *Lr24* and *Lr26*, which inhibit GUS (glucosidase) expression in proximal gene lines, containing the resistance genes *Lr9*, *Lr24*, and *Lr26*, respectively (Segovia et al., 2016). It was also found that Pt18906 acts in the cytoplasm and may cause accumulation of reactive oxygen species (ROS) and callose in TcLr10+27+31 (Qi et al., 2020). We screened and correlatively analyzed *Pt* effector proteins in 2012, and we used the transcriptome of the wheat–*Pt* interaction to perform differential expression analysis and effector protein screening (Wei et al., 2020). A total of 635 candidate secreted effector proteins (CSEPs) were predicted in *Pt* isolates from China through a series of screening methods, including signal peptide prediction, subcellular localization, transmembrane domain prediction, EffectorP, and structural characteristics of CSEPs (Zhang Y et al., 2020). The results of this research laid a foundation for future studies on the molecular mechanisms of host–pathogen interactions, but the biological functions of the identified effectors need to be further studied.

A secreted protein, Pt13024 screened from the 635 CSEPs was significantly up-regulated during the period of *Pt* haustoria formation. To analyze and clarify the role of Pt13024 in the pathogenicity of *Pt*, we cloned the *Pt13024* gene, analyzed the expression profile using quantitative reverse transcription-PCR (qRT-PCR), and also analyzed its ability to inhibit cell death induced by BAX (Bcl-2-associated X protein) and infestan 1 (*Phytophthora infestans* PAMP-INF1). We further analyzed how Pt13024 affects host callose deposition and ROS accumulation by using the bacterial type III secretion assay to transiently express the *Pt13024* in Thatcher and 42 wheat rust-resistant near-isogenic lines (monogenic lines). We also detected the biofunction of Pt13024 in TcLr30 by using the host-induced gene silencing (HIGS) technology. Based on the results of heterologous co-expression on *Nicotiana benthamiana* and on wheat rust-resistant near-isogenic lines, and HIGS analysis, we concluded that Pt13024 is avirulent to TcLr30.

## Materials and methods

### Biological material and growth conditions

The biological materials used in this study includes *N. benthamiana*, Thatcher, single uredium propagules of the *Pt* race 13-5-72 (THSN), which is avirulent to *Lr30*, nine different pathotypes of single uredium propagules of *Pt* (**Supplementary Table 1**), and 42 wheat leaf rust resistant near-isogenic lines (monogenic lines) including TcLr1, TcLr2a, TcLr2b, TcLr2c, TcLr3, TcLr3ka, TcLr3ka, TcLr9, TcLr10, TcLr11, TcLr12, TcLr13, TcLr14a, TcLr14b, TcLr15, TcLr16, TcLr17, TcLr18, TcLr19, TcLr20, TcLr21, TcLr22, TcLr23, TcLr24, TcLr25, TcLr26, TcLr27+31, TcLr28, TcLr29, TcLr30, TcLr32, TcLr33, TcLr34, TcLr34, TcLr35, TcLr37, TcLr38, TcLr41, KS91WGRC11 (*Lr42*), TcLr44, TcLr45, TcLr47, and TcLr51. Wheat seedlings inoculated with *Pt* race 13-5-72 and *N. benthamiana* plants were grown in a greenhouse at 20°C with 16 hours (h) of light and 8 h of darkness. *N. benthamiana* plants aged 4 to 6 weeks were used for the expression analysis.

### Plasmid construction and preparation

**Supplementary Table 2** presents the primers that were used in the construction of plasmids. *Pt13024* was cloned from the cDNA of *Pt* race 13-5-72 (THSN). The first 25 amino acids of *Magnaporthe oryzae*’s Mg87 protein, the *Oomycete* effector Avr1b, and the expected signal peptide sequences of Pt13024 were fused with the vector pSUC2 to evaluate the secretion function. For the purpose of determining the subcellular localization in *N. benthamiana*, the open reading frame (ORF) sequence of Pt13024 minus the signal peptide was introduced into the pGR107 vector. Using the protocol by Sohn et al. (2007), the sequence encoding the mature proteins (minus the putative signal peptide of Pt13024) were cloned into the potato virus X (PVX) vectors pGR107 and pEDV6 for the overexpression of Pt13024 in *N. benthamiana* and wheat. According to Yuan et al. (2011), to silence *Pt13024* in TcLr30, a 267-bp fragment encompassing a portion of the 89-bp untranslated region and a portion of the coding sequence were cloned.

### Analysis of the *Pt13024* gene sequence

*Pt13024* was amplified using the complementary DNA (cDNA) template transcribed from the RNA isolated from Thatcher inoculated with *Pt* (13-5-72). Online software, such as SignalP v5.0 (http://www.cbs.dtu.dk/services/SignalP/), TargetP 2.0 (http://www.cbs.dtu.dk/services/TargetP/), TMHMM 2.0 (transmembrane prediction using hidden Markov models) (http://www.cbs.dtu.dk/services/TMHMM/), Pfam (http://pfam.xfam.org/), the MEME suite (http://meme-suite.org/), and SOPMA (self-optimized prediction method with alignment) (http://npsa-pbil.ibcp.fr/cgi-bin/npsa_automat.pl?Page=npSA_sopma.html) (Jaswal et al., 2020) were employed to analyze the Pt13024 sequence.

### qRT-PCR analysis of *Pt13024*

Ten-day old Thatcher-seedlings were inoculated with *Pt* race 13-5-72 (THSN) at 16°C in the dark, with 100% humidity for 14 h. Total RNA was isolated from the inoculated wheat leaves collected at 6, 12, 18, 24, 36, 48, 72, 96, 144, 216, and 288 h post inoculation of *Pt*. First-strand cDNA was synthesized using a 1 : 10 dilution, and 2 µL of the synthesized cDNA was used for qRT-PCR. The actin gene elongation factor 1 (*EF1*) specific for *Pt* was used as an endogenous reference control to normalize gene expression across different *Pt* samples. Following the method outlined by Wang et al. (2012), the relative gene expression levels of *Pt13024* in the various treatments were assessed by qRT-PCR. The relative gene expression of *Pt13024* was calculated using three independent biological replicates (Livak and Schmittgen, 2012).

### Sequence polymorphism analysis of *Pt13024*

The nine different virulent *Pt* races (09-12-284-1 (THTS), 03-5-99 (PHTP), 04-15-7 (FHRT), 08-5-361-1 (THTT), 08-5-261-2 (THKT), 08-5-9-2 (KHHT), 08-5-11-1 (FHHT), 13-5-28-1 (JHKT), and 13-5-72 (THSN) (**Supplementary Table 1**) were used to inoculate the susceptible wheat Thatcher. The *Pt13024* gene was amplified using the primers (named Pt13024), with the nine different *Pt* races DNA as templates. The PCR products were sequenced by Shanghai Sangon Biotech of China. The software MEGA7 was used for multiple sequence comparison to analyze the polymorphism of candidate effector proteins in the nine isolates.

### Secretory function of Pt13024 signal peptides

The plasmids produced from sucrose symporter 2 (pSUC2) were transformed into yeast strain YTK12 by the lithium acetate technique (Gietz et al., 1995). The SD-Trp medium was used to produce all transformants. Positive colonies were grown on YPRAA plates, which contained raffinose as the carbohydrate source so as to test the production of invertase. The amino acids 1 to 21 of the Pt13024 protein were ligated into pSUC2 vectors. Negative controls included the untransformed YTK12 strain, i.e., the YTK12 strain transformed with an empty pSUC2 vector or the amino acids 1 to 25 of *M. oryzae*’s non-secreted Mg87 protein, whereas positive controls included the recombinant YTK12 strain expressing the signal peptide of Avr1b. At the same time, the control and the positive clones of the gene were cultured in liquid SD-Trp medium. It was washed off with sterilized water after shaking at 220 rpm for 1–2 d, and a phosphate-buffered saline (PBS) solution containing 2% 2,3,5-triphenyltetrazolium chloride (TTC) was added to observe the discoloration.

### Subcellular localization of Pt13024

The Pt13024 protein (with no signal peptide) was cloned and inserted into pGR107:GFP (green fluorescent protein) and transferred into *Agrobacterium tumefaciens* strain GV3101. The *Agrobacterium* strain carrying Pt13024 was diluted to an OD_600_ of 0.2, and pGR107:GFP was used as a control. The transient co-expressions of Pt13024 ΔSP : GFP and GFP alone in leaf tissues of *N. benthamiana* were examined with a Nikon Ti2-U epifluorescence microscope (Nikon Corporation, Japan). Next, the epidermis of *N. benthamiana* leaves was removed and soaked in 0.8M mannitol (Bi et al., 2020), and a Nikon Ti2-U fluorescence microscope (Nikon Corporation, Japan) was used to observe the expression level and localization of effector proteins. GFP autofluorescence was captured using the 488-laser line and the proper emission filter.

### *Agrobacterium*-mediated transient assay

pGR107-Pt13024 was constructed and introduced into the *A. tumefaciens* strain GV3101. Before infiltrating the *N. benthamiana*, PVX : Pt13024 and cell death inducers (BAX and INF1) were diluted to an OD_600_ of 0.3 each and then incubated at room temperature in the dark for 2 h. An *A. tumefaciens* cell suspension containing Pt13024 was originally infiltrated into the site, and *A. tumefaciens* cells carrying BAX/INF1 were infiltrated into the same location 24 h later to assess Pt13024’s inhibition of BAX/INF1-induced cell death. To rule out the possibility of a space-occupying effect of Pt13024 on BAX, the effector protein construct was mixed with BAX or INF1 at a ratio of 1 : 1 before infiltration of the *N. benthamiana*. The symptoms were monitored for 3–5 days (d) after inoculation with BAX/INF1 and photographed 5 d post inoculation. Ethanol was used to decolorize the leaves. *A. tumefaciens* cells carrying GFP or Avr1b were used as negative and positive controls, respectively, and were infiltrated on *N. benthamiana*. Each putative assay was tested on three leaves with three replicates.

### Transient assay of Pt13024 on wheat

A bacterial strain that delivers a putative defense suppressor *via* the type III secretion system is infiltrated into leaves alongside another strain that delivers defense-eliciting effectors in a manner similar to that of Whigham et al. (2015). The pEDV6-Pt13024 vector was constructed and introduced into the *P. fluorescens* strain EtHAn by electroporation (MicroPulser™411BR10634) (Bio-Rad Corporation, United States). PVX : Pt13024 was diluted to an OD_600_ of 1, and *P. syringae* DC3000 was diluted to an OD_600_ of 0.3. The two solutions were mixed in a 1 : 1(V/V) ratio and infiltrated into the first leaves of the 10- to 14-d-old wheat seedlings, and the phenotypes were observed for 3 d post treatment (Ramachandran et al., 2017). Each assay was tested on at least three leaves of different plants and the experiment was repeated three times.

### Analysis of the Pt13024 critical sequence required for suppression of PCD

The sequence of Pt13024 with the partial amino acid deletion was constructed on the PVX vector using the progressive amino acid deletion method (Qi et al., 2019). The mutant genes were inserted into pGR107 and introduced into *Agrobacterium* GV3101. Using the vector primers, PCR amplification was used to confirm specific colonies (**Supplementary Table 2**). The ability of the mutant candidate effector gene to hamper the function of BAX was verified by infiltrating *Agrobacterium* on *N. benthamiana*. The symptoms were monitored for 3 to 5 d after inoculation with BAX/INF1 and photographed 5 d post inoculation. Ethanol was used to decolorize the leaves. *A. tumefaciens* cells carrying GFP or Avr1b were used as negative and positive controls, respectively, and were infiltrated on the *N. benthamiana* strain. Each putative assay was tested on three leaves with three replicates.

### ROS accumulation and callose deposition assays

pEDV6:Pt13024 was constructed and transformed into *P. fluorescens* strain EtHAn by electroporation. The Pt13024 construct was diluted to an OD_600_ of 1.0 and injected into a set of near-isogenic lines with different *Lr* genes. The effects of overexpression of effector proteins on sensitive cells, callose deposition, and ROS accumulation in wheat without or with different resistance genes were observed. For the callose deposition experiment, wheat plants were syringe-infiltrated with EtHAn-Pt13024. The leaves were collected 48 h later and cleaned with 95% (v/v) ethanol. An aniline blue solution containing 0.05% (w/v) was used to stain the fully cleaned leaves. The accumulation of callose deposition was observed under a Nikon Ti2-U fluorescence microscope (Nikon Corporation, Japan), and we calculated the area of callose deposition by using Image-Pro Plus 6.0 software (Ying et al., 2016). By staining with 3,30-diaminobenzidine (DAB) and nitroblue tetrazolium (NBT) dye solution, ROS accumulation was observed. DAB powder (100 mg; Coolaber) was dissolved in 100 ml of water by adding HCl to reach a pH of 3.8. EtHAn-Pt13024 was syringe-injected into wheat plant leaves. The infiltrated leaves were collected 7 d later and left in the DAB solution under light for 12 h. The leaves were then cleaned using 95% ethanol before imaging. We used 100 ml of water to dissolve 100 mg of NBT powder (Coolaber). EtHAn-Pt13024 was syringe-injected into wheat plant leaves. Thereafter, at intervals of 5, 10, 15, 20, 25, and 30 mins, the leaves that had been infected were gathered and soaked in the NBT solution for 12 h. Before imaging, the leaves were cleaned using 95% ethanol. Through the use of Image-Pro Plus 6.0 software, the staining area was determined (Ying et al., 2016), and each treatment was repeated three times.

### BMSV-mediated *Pt13024* gene silencing

*Pt13024* gene silencing was performed using HIGS mediated by the barley stripe mosaic virus (BSMV) according to the method described by Cheng et al. (2017), with slight modifications. After being cloned into pCaBS-bLIC, gene segments without the signal peptide were transformed into the *A. tumefaciens* strain EHA105. The α, β and γ chains of the virus were mixed in a ratio of 1 : 1 : 1 and placed at room temperature for 1 to 3 h before infiltrating into the *N. benthamiana* strain. The infiltrated plants were grown for 10 d in the greenhouse (16:8 h, day : night, at 20°C), after which the infected leaves were harvested, ground in 1% celite in 0.1M PBS solution (at pH 7.4) (Coolaber). The resulting sap was immediately rubbed onto the first leaf of 10-d-old wheat seedlings. Before infecting *N. benthamiana*, the, α, β and γ chains of the virus were combined in a ratio of 1 : 1 : 1 and left at room temperature for 1 to 3 h. The infected *N. benthamiana* plants were grown for 10 d in a greenhouse at a temperature of 20°C for 16 h in daylight and 8 h in darkness. The infected leaves were then plucked and ground in 1% celite in 0.1M PB buffer (at pH 7.4) (Coolaber). With a gloved finger, the resultant sap was immediately applied to the first leaf of 10-d-old wheat seedlings. The treated seedlings were cultured at 20°C for 15 d in the greenhouse, and then a sample of the inoculated *Pt* 13-5-72 race was applied to the cultured seedlings. Samples were collected at 12 hours post inoculation (hpi), 24 hpi, 48 hpi, and 6 days post inoculation (dpi), respectively, and RNA was extracted. The infection type of wheat leaf rust was identified according to the standard of infection type of wheat leaf rust (**Supplementary Table 3**). High-quality RNA was used to detect the silencing efficiency of effector proteins and transcription level fold changes of plant defense-related genes. To observe the infection process of *Pt*, wheat leaves samples were collected at 24 hpi, 48 hpi, and 6 dpi, respectively, and decolorized leaf segments were stained with Fluorescent Brightener 28. Fluorescent Brightener 28 powder (100 mg; Sigma-Aldrich) was dissolved in 100 ml of tris-hydrochloric acid (HCl) buffer solution. An Olympus FV1000 microscope (Olympus Corporation, Japan) was used to examine the stained samples. In this test, three biological duplicates were measured.

## Results

### Expression analysis and structure of *Pt13024*

**Figure 1:**
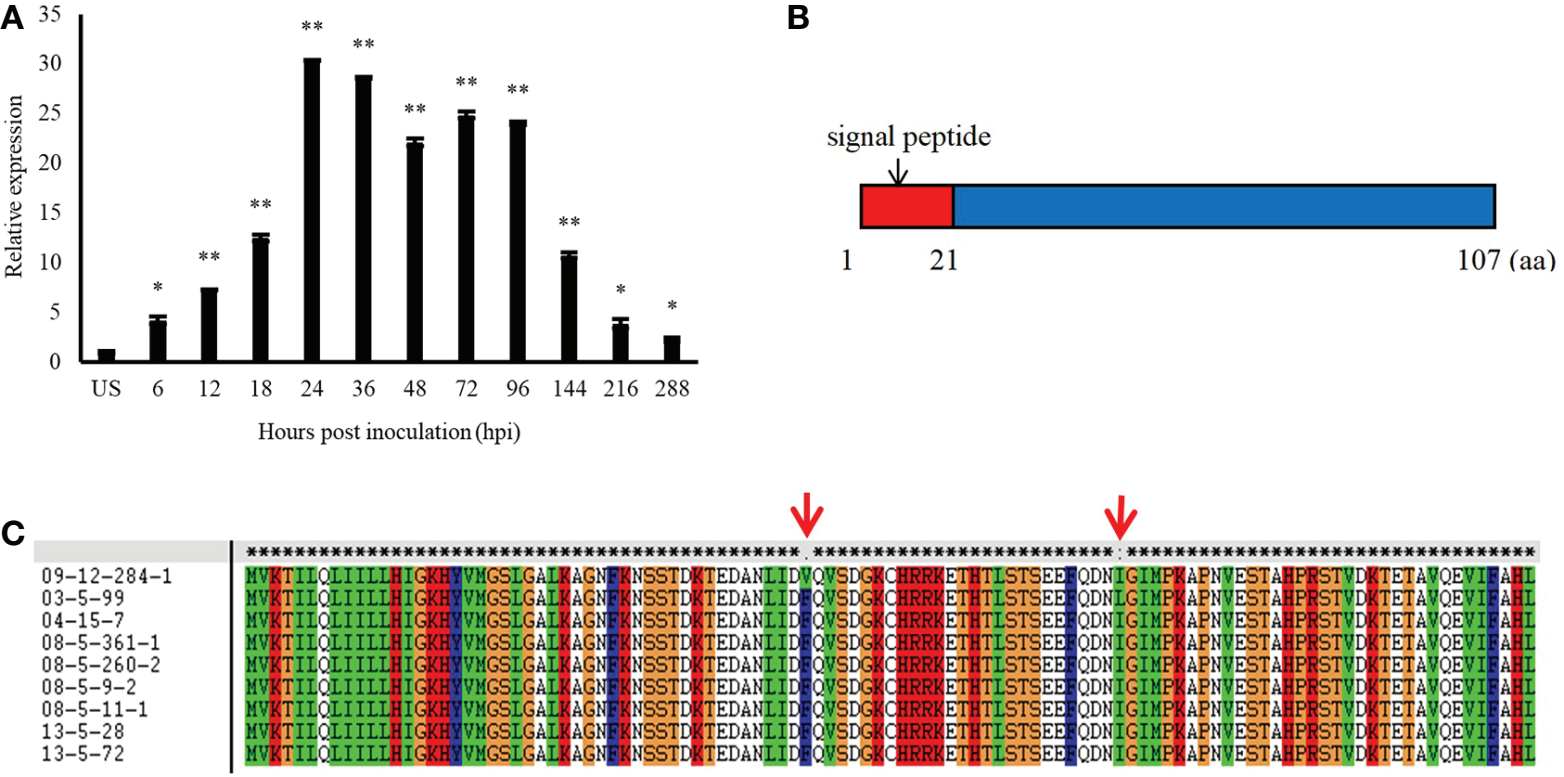
The expression pattern and structure of Pt13024 during the infection of wheat by *P*. **(A)** Transcript levels of *Pt13024* at various stages of *Pt* infection. The relative gene quantification was calculated by the comparative Ct method, with the *Pt*-endogenous gene *EF1* as an internal standard and was relative to that of US. Three technical replicates and biological repetition for each treatment were analyzed. Means and SE from three independent replicates are shown. Asterisks indicate significant differences (**p < 0.01, *p < 0.05). **(B)** The primary structure of Pt13024. **(C)** There were two polymorphic sites of the *Pt13024* gene-encoding protein among nine different *Pt* isolates. The red arrow represents the location of the mutation site. US, urediniospores; *Pt, Puccinia triticina*; SP, signal peptide.

*Pt13024* encodes a secreted protein of 107 amino acids in length, which is typical of fungal effectors, and it is highly expressed during the interaction between *Pt* and wheat. In comparison to the expression level in urediospores, quantitative reverse transcription- polymerase chain reaction (qRT-PCR) analysis showed that the expression level of *Pt13024* was upregulated over 30-fold at 24 hpi, which is the crucial stage of haustorium formation (**Figure 1A**). We analyzed the sequence on the NCBI website and found that it was 100% homologous to the hypothetical protein PTTG_12231. We employed SignalP 4.1, TargetP 2.0, TMHMM 2.0, Pfam, and MEME software to analyze the signal peptide, transmembrane structure, and protein motif, respectively. The analysis results showed that the amino acids from 1 to 21 of the protein were a signal peptide, which is an important feature of the secretion of effectors (**Figure 1B**). We analyzed the Pt13024 secondary structure using the online software SOPMA and found that it had several structural components, including a 29.91% alpha helix, a 16.82% extension chain, a 4.67% angle, and a 48.6% irregular curl. To determine whether or not *Pt13024* is specific to the differential intraspecies of *Pt*, we amplified the target genes from genomic DNA of nine different virulent *Pt* isolates (**Supplementary Table 1**). The analysis of the exons encoding the protein in different isolates showed that there were two polymorphic sites of the Pt13024 protein in nine *Pt* isolates. The 43rd amino acid at the N-terminal was mutated from phenylalanine (F) to valine (V) in 09-12-284-1 (THTS) and the 73rd amino acid was mutated from isoleucine (I) to leucine (L) in 03-5-99 (PHTP) (**Figure 1C**). Pt13024 did not show the deletion or insertion of a single amino acid or large fragments in nine different pathogenic types of *Pt*, suggesting that Pt13024 has a low level of intraspecies polymorphism.

### Secretion validation of the N-terminal signal peptide of Pt13024

**Figure 2:**
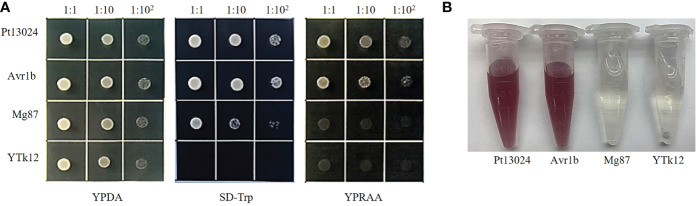
The putative signal peptide of Pt13024 is a functional secretory signal peptide. Functional validation of the putative N-terminal signal peptide of Pt13024 was carried out using the yeast invertase secretion assay and with 2,3,5-triphenyltetrazoliu chloride (TTC). **(A)** The sequence of the putative Pt13024 signal peptide was fused in-frame to the invertase sequence in the pSUC2 vector and then transformed into the yeast strain YTK12. The YTK12 strain, empty pSUC2 vector, and the first 25 amino acids of non-secreted Mg87 proteins from *M. oryzae* were used as negative controls, and the effector Avr1b was used as a positive control. Only the yeast strains that are able to secrete invertase were grown on both SD-Trp and YPRAA media. pSUC2, sucrose symporter 2. **(B)** The secreted invertase activity are detected with 2,3,5-triphenyltetrazoliu chloride (TTC). The cameo brown color indicates invertase activity.

To validate the putative signal peptide of Pt13024’s secretory function, we carried out the genetic assay based on the requirements of yeast cells for invertase secretion to grow on sucrose or raffinose media according to the method of Tian et al. (2011). The predicted signal peptide sequence of *Pt13024* was fused with the vector pSUC2 (Jacobs et al., 1997) and was then transformed into the invertase secretion-deficient yeast strain YTK12 (Oh et al., 2009). The pSUC2 vector and a non-secreted version of pSUC2 where the signal peptide was substituted with the first part of the Mg87 protein from *M. oryzae* (Gu et al., 2011) was used as a negative control, and *P. sojae* effector Avr1b (Shan et al., 2004; Gu et al., 2011) was used as a positive control. The *Pt13024* construct enabled the invertase mutant yeast strain YTK12 to grow on the YPRAA medium (with raffinose instead of sucrose) (**Figure 2A**). This result demonstrated that the putative N-terminal signal peptide of Pt13024 has a valid secretion function. Moreover, we used TTC to detect the enzyme activity (Qi et al., 2019), and we observed the cameo brown color in the reaction tube (**Figure 2B**). These results confirmed the secretion function of the putative N-terminal signal peptide of Pt13024.

### Pt13024 localized to the *N. benthamiana* cytoplasm and nucleus

**Figure 3:**
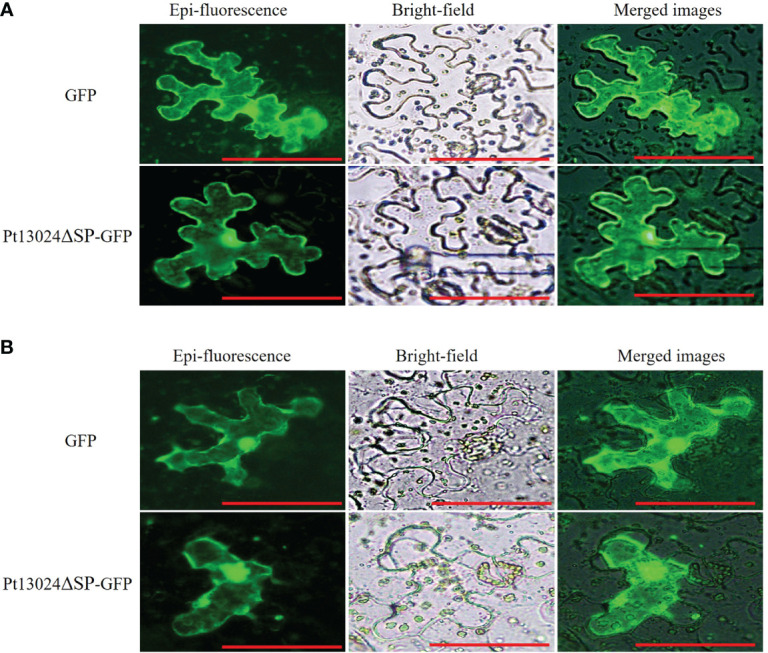
Pt13024 is localized to the *N. benthamiana* cytoplasm and nucleus. **(A)** Transient co-expression of the Pt13024ΔSP : GFP and GFP alone in leaf tissues of *N. benthamiana* was examined using a Nikon Ti2-U epifluorescence microscope (Nikon Corporation, Japan). Bar = 10 μm. **(B)** The epidermis of *N. benthamiana* leaves was removed and soaked in 0.8M mannitol to achieve the purpose of plasmolysis. A Nikon Ti2-U fluorescence microscope (Nikon Corporation, Japan) was used to observe the expression levels and localization of effector proteins. GFP, green fluorescent protein.

After being secreted from pathogens, effectors can be delivered into host plants and target diverse subcellular compartments. To know the subcellular location of Pt13024, we conducted the analysis by using GFP on *N. benthamiana* under a Nikon Ti2-U fluorescence microscope (Nikon Corporation, Japan). The results showed that pGR107:GFP vector and Pt13024–GFP fusion protein were expressed in the nucleus and cytoplasm of the host cell, and green fluorescence was distributed throughout the host cell (**Figure 3A**). After plasmolysis by 0.8M mannitol, it was further observed that the effector protein was located in the cell (**Figure 3B**). This proved that effector protein Pt13024 is localized to the cytoplasm and nucleus.

### Pt13024 suppresses programmed cell death (PPD) in *N. benthamiana* and wheat

**Figure 4:**
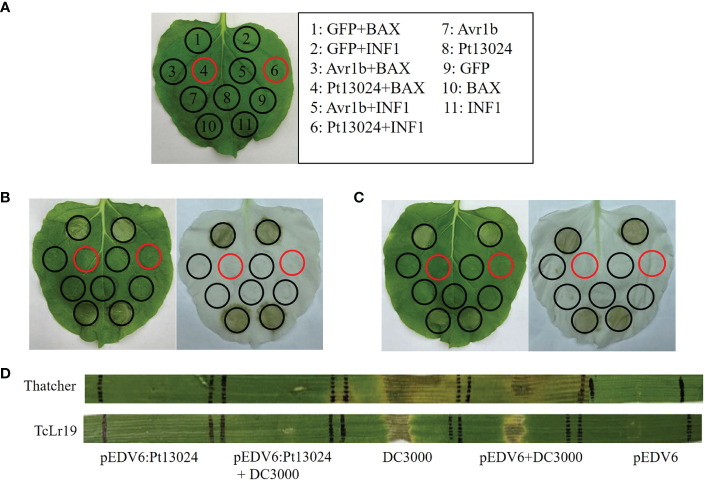
Pt13024 inhibited PCD induced by BAX, INF1, and DC3000. **(A)** The infiltration pattern of the treatments. **(B)** The *Agrobacterium* GV3101 carrying PVX : Pt13024, PVX:eGFP, and PVX : Avr1b were infiltrated in *N. benthamiana*, after 24 hours *Agrobacterium* carrying PVX : BAX or PVX : INF1 were infiltrated on the corresponding positions, respectively. There is no cell death induced by BAX and INF1 24 h after infiltrating with *Agrobacterium* carrying PVX : Pt13024. On the right side is the decolorized phenotype. **(C)** The *N. benthamiana* was infiltrated with 1 : 1 (v/v) mixture of *Agrobacterium* carrying PVX : Pt13024/PVX : GFP/PVX : Avr1b and PVX : BAX/PVX : INF1, respectively. Pt13024 is responsible for the suppression of PCD induced by BAX and INF1. On the right side is the decolorized phenotype. **(D)** Pt13024 delivered *via P. flourescens* EtHAn into leaves of 10- to 14-day- old seedlings of Thatcher and TcLr19, respectively, and suppressed the HR triggered by *P. syringae* pv. *syringae* DC3000. Thatcher and TcLr19 were infiltrated. pEDV6 served as control for co-infiltration experiments. Photographs were taken 3 days after infiltration. BAX, Bcl-2-associated X protein; HR, hypersensitive reaction; PCD, programmed cell death; PVX, potato virus X.

*A. tumefaciens*-mediated transient expression in 4- to 6-week-old *N. benthamiana* was carried out to see whether Pt13024 can inhibit the PCD induced by BAX or INF1. GV3101 strains carrying BAX or INF1 were infiltrated into *N. benthamiana* 24 h post infiltration of pGR107:Pt13024 according to the infiltration pattern diagram (**Figure 4A**). The results showed that GFP, Avr1b, and Pt13024 did not induce the PCD alone. GFP + BAX, BAX, GFP + INF1, and INF1 induced the PCD on *N. benthamiana* leaves. However, there was no necrosis at the infiltration sites of Pt13024 + BAX and Pt13024 + INF1 (**Figure 4B**). This indicated that Pt13024 inhibited the PCD induced by BAX and INF1. We also infiltrated the 1 : 1 (V/V) mixture of BAX or INF1 and Pt13024 on *N. benthamiana* leaves and observed no necrosis at the infiltration sites of Pt13024 + BAX and Pt13024+INF1 (**Figure 4C**). These results show that Pt13024 has the ability to suppress the PCD induced by BAX and INF1.

We tried to convert these findings into a homologous system after discovering the suppressive impact of *Pt* effectors in a heterologous system. Our goal was to deliver a putative suppressor using *P. fluorescens* EtHAn and defense-inducing effectors using *P. syringae* pv. *syringae* DC3000, the bacterium that causes tomato speck. However, when a syringe is inserted into wheat, *P. syringae* DC3000 produces a hypersensitive reaction (HR) (Yin and Hulbert, 2011). To investigate the virulence function of Pt13024 in wheat, we applied the bacterial type III secretion system (T3SS) (Whigham et al., 2015) to deliver a Pt13024 into Thatcher (disease susceptible cultivar) and TcLr19 (disease resistant cultivar), respectively. *P. syringae* DC3000 elicited a HR in both Thatcher and TcLr19. However, pEDV6:Pt13024 and pEDV6 did not trigger a HR in Thatcher and TcLr19 (**Figure 4D**). A HR occurred at the infiltration sites of pEDV6 + DC3000, and there was no significant difference from that of DC3000 alone. The sites of infiltration of pEDV6:Pt13024 + DC3000 produced no lesions on Thatcher or TcLr19. The results indicated that Pt13024 can inhibit the HR induced by DC3000 (**Figure 4D**).

### The amino acids 22–41 at the N-terminal of Pt13024 played a critical role in inhibiting BAX-induced PCD

**Figure 5:**
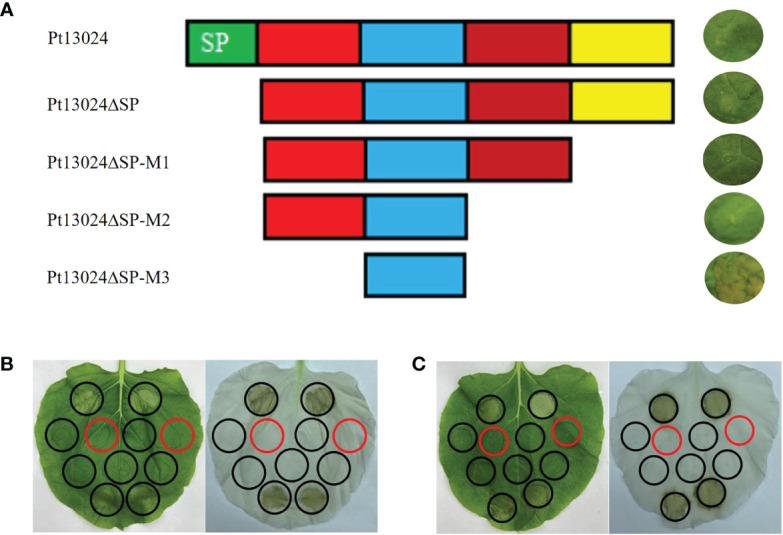
Analysis of the Pt13024 region required for the suppression of PCD. **(A)** The mutants of Pt13024 were constructed and verified on *N. benthamiana.* The toxicity domain of Pt13024 was in the amino acids 22 to 41 at the N-terminal. **(B)** Pt13024 in 09-12-284-1 inhibited PCD induced by BAX and INF1 effectively. On the right is the decolorized phenotype of treated leaf. **(C)** Pt13024 in 03-5-99 strain inhibited PCD induced by BAX and INF1 effectively. On the right is the decolorized phenotype. The infiltration pattern diagram is same as that in **Figure 4A**. aa, amino acid; BAX, Bcl-2-associated X protein; PCD, programmed cell death.

We developed four effector protein Pt13024 deletion mutants to determine the toxic sequence by heterologous expression system. The first mutant (Pt13024△SP) was responsible for the deletion of the signal peptide. The second mutant (Pt13024△SP-M1) was responsible for the deletion of 20 amino acids (△aa 88–108) from the C-terminal. The third mutant (Pt13024△SP-M2) was responsible for the deletion of 40 amino acids (△aa 68–108) from the C-terminal. The fourth mutant (Pt13024△SP-M3) was responsible for the deletion of 20 amino acids (△aa 22–41) at the N-terminal and 40 amino acids (△aa 68–108) at the C-terminal. The results on *N. benthamiana* showed that the mutant without the signal peptides of Pt13024 and the Pt13024 mutant with 20 and 40 amino acid deletions at the C-terminal can still inhibit PCD induced by BAX, but that the Pt13024 mutants with 20 amino acid deletions at the N-terminal (SLGALKAGNFKNSSTDKTED) lost their ability to inhibit PCD. These results suggest that the amino acids 22 to 41 at the N-terminal of Pt13024 are essential for PCD suppression by Pt13024 (**Figure 5A**). There were two polymorphisms of effector protein in the nine *Pt* strains with different virulence, and the polymorphism site was not present in the virulence domain. We transiently expressed them on *N. benthamiana* by *A. tumefaciens* and found that Pt13024 with polymorphisms can still inhibit PCD caused by BAX and INF1 effectively (**Figures 5B, C**). This indicates that the polymorphism of Pt13024 does not affect its inhibitive function on PCD induced by BAX.

### Pt13024 promotes callose deposition and ROS accumulation in TcLr30

**Figure 6:**
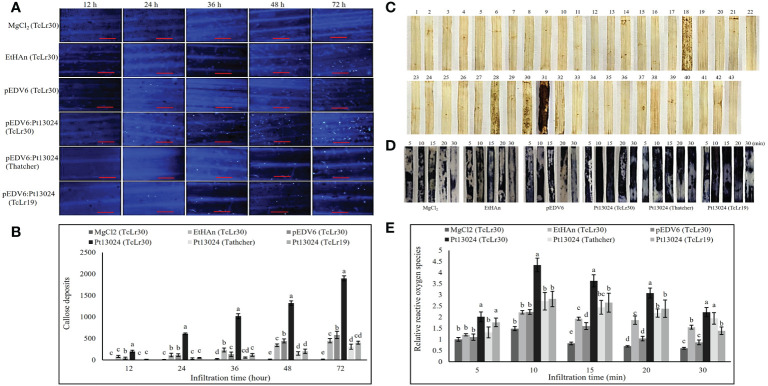
Transient expression of Pt13024 stimulated callose deposition and caused a burst of ROS in TcLr30. **(A)** Callose deposition at different time points after overexpression of Pt13024 in Thatcher, TcLr19, and TcLr30, respectively. The controls were MgCl_2_, EtHAn, and pEDV6. Bar = 100 μm. **(B)** Callose deposition associated with infiltration time. The accumulation of callose deposition was counted by the area using Image-Pro Plus 6.0 software. The area of callose deposition was measured in pixels. **(C)** The effector proteins were delivered into the Thatcher and 42 wheat leaf rust resistant near-isogenic lines (monogenic lines) by the bacterial type III secretion system. The sequence of wheat materials was 1, Thatcher; 2, TcLr1; 3, TcLr2a; 4, TcLr2b; 5, TcLr2c; 6, TcLr3; 7, TcLr3ka; 8, TcLr3bg; 9: TcLr9, 10: TcLr10; 11: TcLr11; 12, TcLr12; 13, TcLr13; 14, TcLr14a; 15, TcLr14b; 16, TcLr15; 17, TcLr16; 18, TcLr17; 19, TcLr18; 20, TcLr19; 21, TcLr20; 22, TcLr21; 23, TcLr22, 24, TcLr23, 25, TcLr24, 26, TcLr25, 27, TcLr26, 28, TcLr27+31, 29, TcLr28; 30, TcLr29, 31, TcLr30, 32, TcLr32, 33, TcLr33, 34, TcLr34, 35, TcLr35, 36, TcLr37; 37, TcLr38, 38, TcLr41, 39, KS91WGRC11 (*Lr42*), 40, TcLr44, 41, TcLr45, 42, TcLr47; and 43, TcLr51. DAB was used to stain, and the phenotypic after decolorization is shown. **(D)** ROS bursts at different time points after overexpression of Pt13024 on Thatcher, TcLr19, and TcLr30. NBT was used to capture O_2_^-^ *in situ* in plant tissue. The controls were MgCl_2_, EtHAn, and pEDV6. **(E)** The ROS after overexpression of Pt13024 for 5, 10, 15, 20, and 30 min were quantified. The staining area was calculated using Image-Pro Plus 6.0 software. The area of ROS was measured in pixels. DAB, 3,30-diaminobenzidine; NBT, nitroblue tetrazolium; ROS, reactive oxygen species.

We expressed Pt13024 in Thatcher (susceptible cultivar) and 42 wheat rust-resistant near-isogenic lines (in Thatcher background) or monogenic lines with different levels of resistance using vector pEDV6 and *P. fluorescens* effector-to-host analyzer (EtHAn) strain to further investigate its role in plant immunity modulation. A high level of callose deposition was found only in TcLr30 at 48 h after infiltration (**Supplementary Figure 1**). We then overexpressed the pEDV6:Pt13024 in TcLr30, susceptible cultivar Thatcher, and resistant wheat near-isogenic line TcLr19. The histological samples were harvested at 0 h, 12 h, 24 h, 36 h, 48 h, and 72 h after infiltration, and observed using a Nikon Ti2-U fluorescence microscope (Nikon Corporation, Japan) (**Figure 6A**). We found that the amount of callose deposition increased gradually (**Figure 6B**). However, the amount of callose deposition in Thatcher or TcLr19 was significantly less than that in TcLr30. We dyed the wheat leaves with DAB and found that Pt13024 can stimulate the accumulation of ROS in the wheat near-isogenic lines TcLr17, TcLr27+31, TcLr30, and TcLr29, but not in others. Comparing the phenotype in TcLr17, TcLr27+31, TcLr30, and TcLr29, we found that Pt13024 stimulated more ROS accumulation in TcLr30 than in TcLr17, TcLr27+31, and TcLr29 (**Figure 6C**). These results indicate that Pt13024 can strongly trigger the host’s defense response. We applied NBT to capture O_2_^-^ *in situ* in TcLr30, Thatcher and TcLr19 tissue and developed a color key, with different colours representing the presence of different elements. The blue color represents the presence of O_2_^-^ (**Figure 6D**). ROS started to accumulate just after infiltration, and peaked at 10 min after infiltration, then decreased. Compared with MgCl_2_, EtHAn and pEDV6, Pt13024 stimulated a larger amount of ROS accumulation (**Figures 6D, E**). This indicates that Pt13024 triggered the TcLr30’s defense response. We wished to find out if Pt13024 could stimulate the accumulation of ROS in other wheat cultivars. To find this out, we overexpressed Pt13024 in Thatcher and TcLr19, which are susceptible and highly resistant to *Pt*, respectively. We observed that Pt13024 greatly stimulated the accumulation of ROS in Thatcher and TcLr19, but that the amount of ROS was still significantly lower than that in TcLr30 (**Figures 6D, E**).

### Silencing of *Pt13024* enhanced the virulence of *Pt* on TcLr30

To investigate the role of *Pt13024* in *Pt* pathogenicity, we used the BSMV-mediated HIGS system in TcLr30 to transiently silence *Pt13024* (Yuan et al., 2011). In the present study, cross-5′-non-coding region and ORF silencing fragments were designed specifically for silencing *Pt13024*. As polymorphism in the gene non-coding region is higher than that of the coding region, the design of silencing fragment across the non-coding region ensures the specificity of silencing. At the same time, the silencing sequence was compared with the NCBI database to ensure that there was no homologous sequence in wheat and *Pt.*

**Figure 7:**
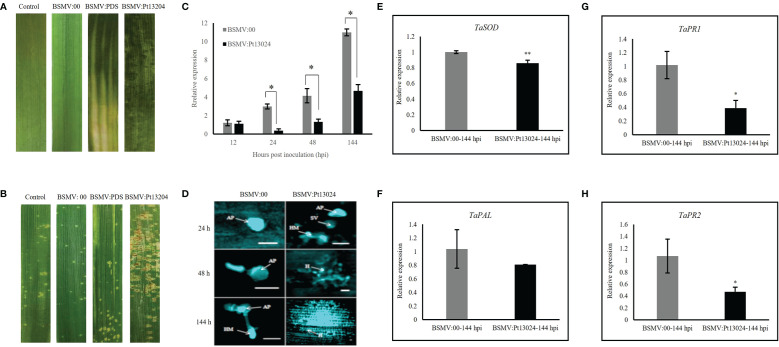
Silencing *Pt13024* increased the virulence of *Pt* 13-5-72 on TcLr30. **(A)** The second leaf of TcLr30 was inoculated with sodium phosphate buffer, barley stripe mosaic virus BSMV:00, and recombinant virus BSMV : PDS and BSMV : Pt13024, which were amplified by *Agroinfiltration-*mediated in *N. benthamiana*. The virus phenotypes on TcLr30 were observed and photographed at 10 dpi. **(B)** TcLr30 seedlings were inoculated with the labeled BSMV constructs on the second leaf for 10 days, and then inoculated with urediospores of *Pt* 13-5-72 (THSN) on the fourth leaf. Leaves infected with *Pt* were investigated at 14 dpi. Infection type “;”: No uredospore pile, but necrosis or chlorosis; “3”: The uredinium is medium with chlorosis around. **(C)** Relative expression of Pt13024 in TcLr30 after inoculation of THSN at 6 d. The transcript levels of Pt13024 genes in leaves infiltrated by virus (BSMV:00) were measured, and inoculated *Pt* was the control. The expression levels the of Pt13024 gene were measured in the leaves infected by the recombinant virus. (*: *p* < 0.05; **: *p* < 0.01) **(D)** Histological observation of wheat TcLr30 infiltrated with BSMV and inoculated *Pt* 13-5-72 (THSN). Bar = 20 µm. **(E–H)** Transcription level fold changes of plant defense-related genes *SOD*, *PAL*, *PR1* and *PR2* in leaves of Wheat silenced with Pt13024. The relative gene quantification was calculated by the comparative Ct method with *Pt* endogenous gene *EF1* as an internal standard. Three technical replicates and biological repetition for each treatment were analyzed. Mean and SE values from three independent replicates are shown. Asterisks indicate significant differences (**p < 0.01, *p < 0.05). AP, appressorium; BSMV, barley stripe mosaic virus; H, haustorium; HMC, haustorial mother cells; IH, infection hyphae; *Pt*, *Puccinia triticina*; SV, substomatal vesicles.

We infiltrated the *Agrobacterium* carrying BSMV:phytoene desaturase (PDS) and BSMV : Pt13024 into *N. benthamiana*. Obvious photobleaching was observed in the BSMV : PDS-inoculated plants that had the wheat PDS gene silenced at 10 dpi (**Figure 7A**). These results indicated that the HIGS system was effective. The BSMV:00 (mock) and BSMV : Pt13024-infiltrated wheat plants were then inoculated with *Pt* isolate 13-5-72 (avirulence to *Lr30*), and the phenotypes were photographed at 14 dpi with *Pt*. The results showed that the symptoms of mock and BSMV : PDS were similar to those in the control leaves infiltrated with PBS buffer solution (**Figure 7A**), indicating that BSMV had no effect on the interaction between wheat and *Pt*. 14 d after inoculation of 13-5-72 (THSN) on the infiltrated TcLr30 with BSMV : Pt13024, the infection phenotype of *Pt* on TcLr30 changed from “;” to “3” (**Figure 7B**). qRT-PCR analysis showed that the transcript level of *Pt13024* was significantly reduced, but not eliminated in the BSMV : Pt13024-infiltrated wheat plants compared with the control (**Figure 7C**), suggesting that *Pt13024* was partially silenced by HIGS. We observed germinated spores, germ tube and appressorium of *Pt* on TcLr30 at 24 hpi of BSMV:00, and the haustoria mother cells, but no haustorium was observed at 6 dpi under an Olympus FV1000 microscope (Olympus Corporation, Japan). *Pt* appressorium, substomatal vesicles, infection hypha, and haustoria mother cells were observed at 24 hpi after infiltrating BSMV : Pt13024 on TcLr30, and a large number of haustoria and hyphae were observed at 6 dpi (**Figure 7D**). qRT-PCR was conducted to evaluate the expression levels of four genes which were confirmed to be related to wheat immune responses: *SOD*, *PAL*, *PR1*, and *PR2*. When *Pt13024* was silenced, the expression levels of *SOD*, *PAL*, *PR1*, and *PR2* were reduced. These results indicated that *Pt13024* is able to promote the expression of PTI-associated marker genes (**Figures 7E–H**), and that the silencing of *Pt13024* effectively promotes the infection of *Pt* and its development in wheat cells. We conclude that *Pt13024* is avirulent to TcLr30.

## Discussion

As a biotrophic fungus, the leaf rust pathogen can complete infection of its host by forming haustoria, which are essential for nutrient acquisition from the host and delivery of secreted effectors into the host cells. Researchers have identified a variety of effector proteins in different fungi, and these identified effector proteins are mostly secreted proteins with unknown functions (Cuomo et al., 2017). In this study, we analyzed the amino acid sequence and structure of Pt13024 from *Pt* and found that Pt13024 is a small molecule with 107 amino acids that contains a signal peptide (**Figure 1B**), but does not contain a mitochondrial sequence, transmembrane structure, or a conserved motif. The expression level of this gene was higher at 24 hpi than at other time points (**Figure 1A**). It can inhibit cell death induced by BAX and INF1 (**Figures 4B, C**). Candidate secreted effector proteins are selected based on the following criteria: (1) no transmembrane domain, except at the signal peptide region; (2) small size, of less than 300 amino acids; (3) no known conserved Pfam domain; and (4) high levels of messenger RNA (mRNA) expression in the infection cycle or haustoria (Sperschneider et al., 2018; Xu et al., 2020). Pt13024 possesses all the qualities of an effector protein; hence, we concluded that Pt13024 is an effector protein.

There are different key functional amino acid sequences among different effector proteins. In another study of the *Pst*, *PstGSRE1* was transiently expressed in *N. benthamiana* using agroinfiltration, and it was found that a small region covering amino acids 91 to 140 (PstGSRE1-m9) was sufficient to suppress PCD (Qi et al., 2019). The immunosuppressive activity of the effector SCRE1 is restricted to a short peptide sequence that contains the crucial “cysteine–proline–alanine–arginine–serine” motif (Zhang N et al., 2020). In this study, we found that the mutant with 20 amino acids missing at the N-terminal, that is, amino acids 22 to 41, lost its PCD suppression activity (**Figure 5A**), suggesting that the N-terminal with amino acids 22 to 41 (SLGALKAGNFKNSSTDKTED) plays a key role in the function of effector protein Pt13024. The reason why the functional region is at the N-terminal ought to be further studied, and it is also important that the specific amino acid sequence there is further revealed.

The plant defense system consists of both PAMP-triggered immunity (PTI) and effector-triggered immunity (ETI) defense systems. The deposition of callose indicates that PTI has occurred in plants. The death of host cells may also occur during this process, but the response is less intense than that of ETI. As the second defense system, ETI is mainly activated by the NB-LRR type of disease-resistant proteins that directly or indirectly recognize the effectors secreted into plant cells by pathogenic microorganisms, and triggers a stronger immune response. ETI tends to produce large amounts of peroxides such as hydrogen peroxide (H_2_O_2_) in the infected host cells, resulting in HR induction. PstGSRE1 suppressed ROS-mediated cell death and compromised host immunity (Qi et al., 2019). To learn more about the role of Pt13024 in modulating host immunity, we overexpressed it in Thatcher and 42 wheat leaf rust resistant near-isogenic lines (monogenic lines) with various resistance genes. The results showed that Pt13024 induced the most callose deposition and ROS accumulation in TcLr30 than in others. Moreover, the callose deposition increased with the extension of effector overexpression time. The ROS burst occurred before callose deposition. ROS is a single-electron reduction product of an oxygen class. We used NBT to detect O_2_^-^ and we observed H_2_O_2_ after expressing effector protein on wheat for 5 d. The results indicated that Pt13024 does not only trigger PTI, but also ETI.

HIGS is an effective technology for studying gene functions, and it is widely used in the study of pathogenic genes of biotrophic plant fungal pathogens such as *Pst*, *Pt* and *Erysiphe pisi*. Zhao et al. (2018) employed BSMV-mediated HIGS to silence the expression of *Pst8713*. The results demonstrated that silencing *Pst8713* changes the *Pst* pathogenicity phenotype, revealing *Pst8713*’s role in *Pst* virulence. We used HIGS to silence the effector protein *Pt13024* during the interaction between the avirulence *Pt* race 13-5-72 and *Lr30*. qRT-PCR expression analysis showed that the expression of *Pt13024* reached its peak at 24 hpi, which is an important period for *Pt*’s formation of haustoria. In combination with the expression pattern of *Pt13024* in infected wheat, we speculated that *Lr30* can inhibit the formation of haustoria by recognizing Pt13024 when the pathogen infects TcLr30. So, the target proteins before and after gene silencing should be analyzed in the future. All the results above indicated that Pt13024 triggered the strongest resistance in TcLr30. In addition, Pt13024 can stimulate a small amount of ROS in TcLr17, TcLr27+31, and TcLr29. Besides the study on callose deposition and ROS accumulation caused by Pt13024, silencing of *Pt13024* on TcLr30, we will undertake other relevant studies on TcLr17, TcLr27+31, and TcLr29 in future.

Segovia et al. (2016) found that Pt3 and Pt27 inhibited GUS in near-isogenic lines containing resistance genes *Lr9*, *Lr24*, and *Lr26*, respectively, and it was speculated that they had avirulent effects on *Lr9*, *Lr24* and *Lr26*. In this study, we found that Pt13024 induced ROS accumulation and callose deposition in TcLr30, and it was higher than others when Pt13024 was overexpressed in susceptible wheat Thatcher and the 42 wheat leaf rust resistance near-isogenic lines (monogenic lines) with different resistance genes. Silencing of *Pt13024* resulted in the successful infection of TcLr30 by 13-5-72 (THSN) which is a strain of avirulence race to TcLr30. Therefore, it is speculated that the effector protein has an avirulent effect on TcLr30. This study lays a foundation for revealing the pathogenicity mechanisms of *Pt* effector proteins. In addition, there are many questions that need to be answered, such as: “how the effector protein Pt13024 triggers its host’s immune response?”; “what is the interaction target in the host plant?”; and “which mode of interaction between effector and host target,and what is the function of the target?”.

## Conclusion

Pt13024 is a small protein of 107 amino acids residues. It acts in host cells and the signal peptide has secretory activity. Pt13024 effectively inhibited the PCD induced by BAX and INF1. The amino acids 22 to 41 at the N-terminal play an important role in PCD inhibition. This protein can trigger the strongest resistance reaction including the large amount of callose deposition; the strongest ROS burst occurred in TcLr30 and resulted in a HR. The silencing of *Pt13024* enhanced the virulence of *Pt* 13-5-72 (THSN) in TcLr30. The effector protein confers avirulence to TcLr30.

## Data availability statement

The datasets presented in this study can be found in online repositories. The names of the repository/repositories and accession number(s) can be found below: https://www.ncbi.nlm.nih.gov/genbank/, Pt13024 (MW413286).

## Author contributions

YQ, JL, and WY contributed to the design of the work. YQ, JC, and YZ performed the experiments. YQ, JW, and LC analyzed the sequencing data. YQ wrote the manuscript and JM revised the manuscript. YQ, JM, NZ, DL, QS, and WY drafted the manuscript. All authors read and approved the submitted version.

## Supplementary material

Supplementary Figure 1 Transient expression of Pt13024 stimulated callose deposition in TcLr30. The effector protein was delivered to the Thatcher and 42 wheat varieties with different resistance genes (mongenic lines) by the bacteria type III secretion assay. The callose deposition was observed after overexpression of Pt13024 at 48 hours. 1–43 represent Thatcher, TcLr1, TcLr2a, TcLr2b, TcLr2c, TcLr3, TcLr3ka, TcLr3bg, TcLr9, TcLr10, TcLr11, TcLr12, TcLr13, TcLr14a, TcLr14b, TcLr15, TcLr16, TcLr17, TcLr18, TcLr19, TcLr20, TcLr21, TcLr22, TcLr23, TcLr24, TcLr25, TcLr26, TcLr27 +31, TcLr28, TcLr29, TcLr30, TcLr32, TcLr33, TcLr34, TcLr35, TcLr37, TcLr38, TcLr41, KS91WGRC11 (*Lr42*), TcLr44, TcLr45, TcLr47, and TcLr51, respectively. Bar = 100 μm.
