# Supplementary figures and images for: Wheat leaf rust fungus effector Pt13024 is avirulent to TcLr30
Source: Front Plant Sci. 2023 Jan 16;13:1098549. doi: 10.3389/fpls.2022.1098549 (PMC9885084; doi:10.3389/fpls.2022.1098549)

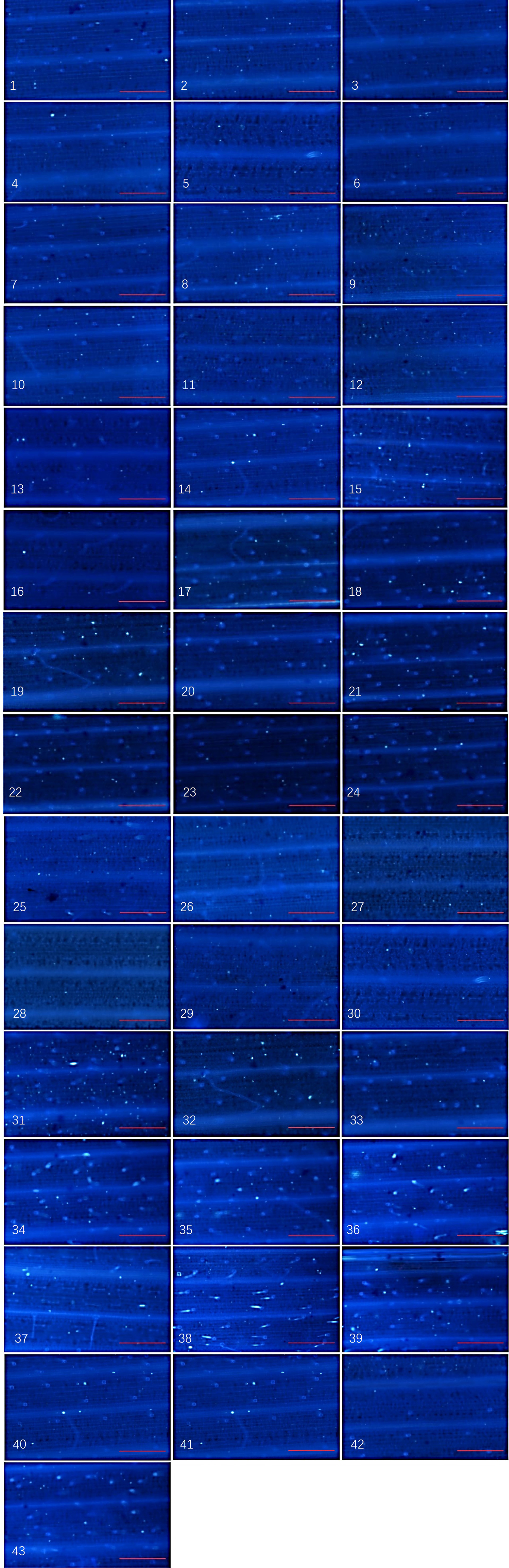

Supplement: Supplementary Figure 1 — Transient expression of Pt13024 stimulated callose deposition in TcLr30. The effector protein was delivered to the Thatcher and 42 wheat varieties with different resistance genes (mongenic lines) by the bacteria type III secretion assay. The callose deposition was observed after overexpression of Pt13024 at 48 hours. 1–43 represent Thatcher, TcLr1, TcLr2a, TcLr2b, TcLr2c, TcLr3, TcLr3ka, TcLr3bg, TcLr9, TcLr10, TcLr11, TcLr12, TcLr13, TcLr14a, TcLr14b, TcLr15, TcLr16, TcLr17, TcLr18, TcLr19, TcLr20, TcLr21, TcLr22, TcLr23, TcLr24, TcLr25, TcLr26, TcLr27 +31, TcLr28, TcLr29, TcLr30, TcLr32, TcLr33, TcLr34, TcLr35, TcLr37, TcLr38, TcLr41, KS91WGRC11 (Lr42), TcLr44, TcLr45, TcLr47, and TcLr51, respectively. Bar = 100 μm. [file Image_1.jpeg]
