# Supplementary material for: Wheat leaf rust fungus effector Pt13024 is avirulent to TcLr30
Source: Front Plant Sci. 2023 Jan 16;13:1098549. doi: 10.3389/fpls.2022.1098549 (PMC9885084; doi:10.3389/fpls.2022.1098549)
Supplement: Supplementary file 2 [file Table_1.docx]

| Strain number | Pathotype | Virulence formula |
| --- | --- | --- |
| 09-12-284-1 | （THTS） | *(Lr1*, *Lr9*, *Lr18*, *Lr19*, *Lr24*, *Lr38*, *Lr42*, *Lr47)/(Lr2a*, *Lr2b*, *Lr2c*, *Lr3*, *Lr3bg*, *Lr3ka*, *Lr10*, *Lr11*, *Lr14a*, *Lr14b*, *Lr15*, *Lr16*, *Lr1*7, *Lr21*, *Lr23*, *Lr25*, *Lr26*, *Lr28*, *Lr29*, *Lr30*, *Lr32* ,*Lr33*, *Lr36*, *Lr37*, *Lr44*, *Lr45*, *Lr46*, *Lr50)* |
| 03-5-99 | (PHTP) | (*Lr2a*, *Lr9*, *Lr10*, *Lr19*, *Lr23*, *Lr24*, *Lr36*, *Lr38*, *Lr42*, *Lr47*)/(*Lr1*, *Lr2b*, *Lr2c*, *Lr3, Lr3bg*, *Lr3ka*, *Lr11*, *Lr14a*, *Lr14b*, *Lr15*, *Lr16*, *Lr17*, *Lr18*, *Lr21, Lr25, Lr26*, *Lr28*, *Lr29*, *Lr30*, *Lr32*, *Lr33*, *Lr37*, *Lr44*, *Lr45*, *Lr46*, *Lr50*) |
| 04-15-7 | (FHRT) | *(Lr1*, *Lr2a*, *Lr9*, *Lr14a*, *Lr15*, *Lr17*, *Lr19*, *Lr23*, *Lr24*, *Lr32*, *Lr36*, *Lr38*, *Lr42*, *Lr46*, *Lr47*)*/*(*Lr2b*, *Lr2c*, *Lr3*, *Lr3bg*, *Lr3ka*, *Lr10*, *Lr11*, *Lr14b*, *Lr16*, *Lr18*, *Lr21*, *Lr25*, *Lr26*, *Lr28*, *Lr29*, *Lr30*, *Lr33*, *Lr37*, *Lr44*, *Lr45*, *Lr50)* |
| 08-5-361-1 | (THTT) | (*Lr1*, *Lr9*, *Lr19*, *Lr14a*, *Lr24*, *Lr36*, *Lr38*, *Lr42*, *Lr44*, *Lr47*) */*(*Lr2a*, *Lr2b*, *Lr2c*, *Lr3*, *Lr3bg*, *Lr3ka*, *Lr10*, *Lr11*, *Lr14b*, *Lr15*, *Lr16*, *Lr17*, *Lr18*, *Lr21*, *Lr23*, *Lr2*5, *Lr26*, *Lr28*, *Lr29*, *Lr30*, *Lr32*, *Lr33*, *Lr37*, *Lr45*, *Lr46*, *Lr50*) |
| 08-5-260-2 | (THKT) | (*Lr1*, *Lr2a*, *Lr9*, *Lr19*, *Lr23*, *Lr24*, *Lr32*, *Lr36*, *Lr38*, *Lr42*, *Lr45*, *Lr46*, *Lr47*)/(*Lr2b*, *Lr2c*, *Lr3*, *Lr3bg*, *Lr3ka*, *Lr10*, *Lr11*, *Lr14a*, *Lr14b*, *Lr15*, *Lr16*, *Lr17*, *Lr18*, *Lr21*, *Lr25*, *Lr26*, *Lr28*, *Lr29*, *Lr30*, *Lr33*, *Lr37*, *Lr44*, *Lr50*) |
| 08-5-9-2 | (KHHT) | (*Lr1*, *Lr2a*, *Lr3ka*, *Lr9*, *Lr15*, *Lr17*, *Lr19*, *Lr23*, *Lr24*, *Lr32*, *Lr36*, *Lr38*, *Lr46*, *Lr47*)/(*Lr2b*, *Lr2c*, *Lr3*, *Lr3bg*, *Lr10*, *Lr11*, *Lr14a*, *Lr14b*, *Lr16*, *Lr18*, *Lr21*, *Lr25*, *Lr26*, *Lr28*, *Lr29*, *Lr30*, *Lr33*, *Lr37*, *Lr42*, *Lr44*, *Lr45*, *Lr50*) |
| 08-5-11-1 | (FHHT) | (*Lr1*, *Lr2a*, *Lr3ka*, *Lr9*, *Lr15*, *Lr17*, *Lr19*, *Lr23*, *Lr24*, *Lr32*, *Lr36*, *Lr38*, *Lr46*, *Lr47*)/(*Lr2b*, *Lr2c*, *Lr3*, *Lr3bg*, *Lr10*, *Lr11*, *Lr14a*, *Lr14b*, *Lr16*, *Lr18*, *Lr21*, *Lr25*, *Lr26*, *Lr28*, *Lr29*, *Lr30*, *Lr33*, *Lr37*, *Lr42*, *Lr44*, *Lr45*, *Lr50*) |
| 13-5-28-1 | (JHKT) | (*Lr1*, *Lr3*, *Lr3bg*, *Lr3ka*, *Lr9*, *Lr15*, *Lr19*, *Lr24*, *Lr38*, *Lr47*)/(*Lr2a*, *Lr2b*, *Lr2c*, *Lr10*, *Lr11*, *Lr14a*, *Lr14b*, *Lr16*, *Lr17*, *Lr18*, *Lr21*, *Lr23*, *Lr25*, *Lr26*, *Lr28*, *Lr29*, *Lr30*, *Lr32*, *Lr33*, *Lr36*, *Lr37*, *Lr42*, *Lr44*, *Lr45*, *Lr46*, *Lr50*) |
| 13-5-72 | (THSN) | *(Lr1*, *Lr2a*, *Lr9*, *Lr19*, *Lr24*, *Lr30*, *Lr32*, *Lr36*, *Lr38*, *Lr42*, *Lr44*, *Lr45*, *Lr46*, *Lr47)/(Lr2b*, *Lr2c*, *Lr3*, *Lr3bg*, *Lr3ka*, *Lr10*, *Lr11*, *Lr14a*, *Lr14b*, *Lr15*, *Lr16*, *Lr17*, *Lr18, Lr21*, *Lr23*, *Lr25*, *Lr26*, *Lr28*, *Lr29*, *Lr33*, *Lr37*, *Lr50)* |

**TABLE S1** The virulence formula of the 9 wheat leaf rust single spore produce.
