# Supplementary material for: Wheat leaf rust fungus effector Pt13024 is avirulent to TcLr30
Source: Front Plant Sci. 2023 Jan 16;13:1098549. doi: 10.3389/fpls.2022.1098549 (PMC9885084; doi:10.3389/fpls.2022.1098549)
Supplement: Supplementary file 3 [file Table_2.docx]

| Primer type | Primer name | Sequence（5’——3’） | Sequence length/bp |
| --- | --- | --- | --- |
| open reading frame  Signal peptide  Subcellular localization  Transient expression  Pt13024 mutation  Type III secretion system  HIGS  qRT-PCR  Defense-related genes | Pt13024-F  Pt13024-R  SPPt13024-F  SPPt13024-R  DPt13024-F  DPt13024-R  TPt13024-F  △SP Pt13024-F  TPt13024-R  LBA  LBB  qPt13024-F1  qPt13024-R1  qPt13024-R2  PPt13024-F  PPt13024-R  H13024-F  H13024-R  RT13024-F  RT13024-R  EFI-F  EFI-R  TaSOD-F  TaSOD-R  TaPAL-F  TaPAL-R  TaPR1-F  TaPR1-R  TaPR2-F  TaPR2-R | ATGGTCAAAACTATTCTCCA  TCACAAATGAGCGAAGATTA  CCGGAATTCATGGTCAAAACTATTCTCCA  CCGCTCGAGTCCCATAACGTAGTGCTTCC  CCCATCGATAGCTTGGGGGCTTTGAAAGC  TCCCCCGGGCAAATGAGCGAAGATTACTT  TCCCCCGGGATGGTCAAAACTATTCTCCA  TCCCCCGGGAGCTTGGGGGCTTTGAAAGC  GCGTCGACTCACAAATGAGCGAAGATTA  CAATCACAGTGTTGGCTTGC  GACCCTATGGGCTGTGTTG  CCCATCGATGCGAATTTGATTGATTTTCA  TCCCCCGGGATGAGCAGTGCTTTCTACGT  TCCCCCGGGTTCAGATGTCGATAGGGTAT  CACCATGGTCAAAACTATTCTCCA  TCACAAATGAGCGAAGATTA  AAGGAAGTTTAAGTTTTCTTTGTGTTGAGC  AACCACCACCACCGTTTCTGTTTTATCAGTCGA  CAAGTGCCACAGACGAA  TCACAAATGAGCGAAGA  TGGTCGTTTTGCTGTGAGGG  GCAGCCTTGGTCACCTTTG  CCGAGGTCTGGAACCATCAC  AGCCGAAATCCTTCTCGATCT  TTCGATTTGCCACCAAGTC  GTGCCTTGGAAGTTGCCAC  GAGAATGCAGACGCCCAAGC  CTGGAGCTTGCAGTCGTTGATC  AGGATGTTGCTTCCATGTTTGCCG  AAGTAGATGCGCATGCCGTTGATG | 20  20  29  29  29  29  29  29  28  21  20  29  29  29  24  20  30  33  17  17  20  19  20  21  19  19  21  22  24  25 |

**TABLE S2** Primers used in this study.
