# Supplementary material for: Wheat leaf rust fungus effector Pt13024 is avirulent to TcLr30
Source: Front Plant Sci. 2023 Jan 16;13:1098549. doi: 10.3389/fpls.2022.1098549 (PMC9885084; doi:10.3389/fpls.2022.1098549)
Supplement: Supplementary file 4 [file Table_3.docx]

**TABLE S3** The standard of infection type of wheat leaf rust.

| Infective type | Symptom |
| --- | --- |
| 0 | No uredospore pile or infection spots visible to the naked eye |
| ; | No uredospore pile, but necrosis or chlorosis |
| 1 | The uredospore pile is small, and there are necrotic spots around |
| 2 | The uredospore pile is small to medium, with necrosis or chlorosis around |
| 3 | The uredospore pile is medium, and sometimes there is chlorosis around |
| 4 | The uredospore pile is large, without chlorosis and necrosis |
